# Ascochlorin Attenuates the Early Stage of Adipogenesis via the Wnt/β-Catenin Pathway and Inhibits High-Fat-Diet-Induced Obesity in Mice

**DOI:** 10.3390/ijms251810226

**Published:** 2024-09-23

**Authors:** Mi-Hee Yu, Yun-Jeong Jeong, Sung Wook Son, So Yoon Kwon, Kwon-Ho Song, Ho-Sang Son, Eon-Ju Jeon, Young-Chae Chang

**Affiliations:** 1 Research Institute of Biomedical Engineering and Department of Cell Biology, Daegu Catholic University School of Medicine, Daegu 42472, Republic of Korea; 2 Department of Internal Medicine, Daegu Catholic University School of Medicine, Daegu 42472, Republic of Korea; 3 Department of Internal Medicine, Raphael Hospital, Daegu 41968, Republic of Korea

**Keywords:** ascochlorin, adipogenesis, β-catenin, high-fat diet, obesity

## Abstract

This study investigated the effects of ascochlorin (ASC), a natural compound derived from the fungus *Ascochyta viciae*, on adipogenesis and obesity. We determined the effects of ASC on 3T3-L1 preadipocytes and whether it ameliorated to mitigate high-fat diet (HFD)-induced obesity in C57BL/6J mice. We found that ASC significantly inhibited the differentiation of preadipocytes by modulating the Wnt/β-catenin signaling pathway, a key regulator of adipogenic processes. Treatment with ASC not only reduced the mRNA and protein expression of key adipogenic transcription factors such as C/EBPα and PPARγ, but also reduced lipid accumulation both in vitro and in vivo. In addition, treatment HFD-fed mice with ASC significantly reduced their weight gain and adiposity vs. control mice. These results suggest that ASC has considerable potential as a therapeutic agent for obesity, owing to its dual action of inhibiting adipocyte differentiation and reducing lipid accumulation. Thus, ASC represents a promising candidate as a natural anti-obesity agent.

## 1. Introduction

Obesity is a global health problem, and it significantly increases the risk of various metabolic diseases, including type 2 diabetes, cardiovascular disease, and certain cancers [1]. The etiology of obesity is complex, involving genetic, environmental, and behavioral factors [2]. One of the critical processes in the development of obesity is the differentiation of preadipocytes into mature adipocytes, a process known as adipogenesis [3]. The expression of PPAR-γ and C/EBPα plays a crucial role in the transition from preadipocytes to adipocytes [4,5] and is regulated by SREBP-1c, which promotes the synthesis of triglycerides from excess energy-rich substances and stores them in adipose tissue and the liver [6,7]. By targeting the signaling pathways that regulate these key players in adipogenesis within adipocytes, it is possible to inhibit triglyceride synthesis and ameliorate obesity.

Adipogenesis is regulated by several signaling pathways, including the Wnt/β-catenin pathway, which plays a pivotal role by inhibiting the differentiation of preadipocytes into adipocytes [8]. The activation of Wnt signaling in preadipocytes reduces the mRNA and protein expression of key transcription factors, such as C/EBPα and PPARγ, thereby inhibiting adipogenesis [9]. This inhibition prevents the accumulation of lipid droplets within cells, which are a hallmark of mature adipocytes [10]. Another important phase in adipocyte differentiation is mitotic clonal expansion (MCE), during which preadipocytes re-enter the cell cycle and undergo several rounds of cell division before they achieve terminal differentiation [11]. MCE is essential for adipogenic gene expressions and the progression of adipogenesis [12], and, therefore, the appropriate regulation of MCE is necessary for the successful differentiation of preadipocytes into adipocytes.

Ascochlorin (ASC) was originally isolated and purified from the plant-specific fungus *Ascochyta viciae*. It is a multifunctional natural compound that features a trimethyl oxocyclohexyl component in its chemical backbone [13]. ASC and its derivatives exhibit a wide array of therapeutic effects, including antiviral, antifungal, anti-hypertension, immunomodulatory, and antidiabetic effects, indicating that it has pharmacologic potential in a number of areas [14,15,16,17]. Although various biologic effects of ASC have been studied, including its antibiotic, antifungal, and anti-tumor effects, its potential anti-obesity effects have not been evaluated to date.

In this study, we aimed to determine the effect of ASC on adipogenesis using 3T3-L1 preadipocytes and explored its potential to prevent high-fat diet (HFD)-induced obesity in vivo. Furthermore, we aimed to elucidate the molecular mechanisms underlying the effects of ASC, specifically focusing on its impact on the Wnt/β-catenin pathway and the expression of key adipogenic and anti-adipogenic genes. We found that ASC inhibits adipogenesis and reduces lipid accumulation in vitro, and attenuates HFD-induced obesity in vivo. These results suggest that ASC may represent a promising means of preventing and/or managing obesity.

## 2. Results

### 2.1. ASC Inhibits Lipid Accumulation in 3T3-L1 Preadipocytes

**Figure 1:**
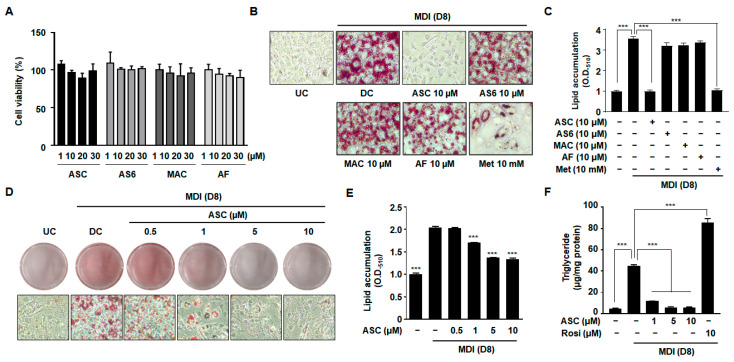
Effect of various compounds on lipid accumulation, TG content, and cell viability in 3T3-L1 adipocytes. (**A**) Two-day post-confluency preadipocytes were incubated with differentiation medium in the presence of various compounds (0, 1, 10, 20, and 30 µM) for 48 h. Cell viability was detected by MTT. (**B**,**D**) Accumulation of intracellular lipid droplets in Oil Red O staining of the undifferentiated control (UC) and differentiated control (DC) or compound-treated cells on day 8 (D8) after differentiation induction. Metformin (Met 10 mM) was used as a positive control. 3T3-L1 cells were imaged using a microscope (×200). (**C**,**E**) Quantification of intracellular lipid content (O.D._510_). (**F**) Measurement of intracellular triglyceride (TG) content on D8. Rosiglitazone (Rosi 10 µM) was used as a negative control. Data represent the mean ± SD of three independent experiments. *** *p* < 0.001 compared to the DC.

To determine whether ASC and its derivatives, 4-O-carboxymethylascochlorin (AS-6) and 4-O-methylascochlorin (MAC), have toxic effects in 3T3-L1 cells, a cell viability assay was conducted. There was no obvious reduction in cell viability after treatment with ASC or its derivatives at concentrations ranging from 1 to 30 µM (Figure 1A). The 3T3-L1 preadipocytes were incubated in MDI medium (insulin, IBMX, and dexamethasone) containing 10 µM ASC or its derivatives, and 8 days later, the cultured 3T3-L1 cells had differentiated into adipocytes and accumulated lipid in droplets in their cytoplasm. The degree of differentiation of the 3T3-L1 preadipocytes into lipid-laden adipocytes was assessed using Oil Red O staining to identify lipid accumulation (Figure 1B,C). Compared with the differentiated control (DC) cells, no significant effects on lipid accumulation were observed in cells treated with AS-6, MAC, or ascofuranone (AF). By contrast, treatment with 10 µM ASC significantly decreased the formation of lipid droplets with higher efficacy compared to treatment with metformin (Met), a positive control involved in inhibiting adipocyte differentiation. We evaluated the antiadipogenic effects of ASC at concentrations of 0.5, 1, 5, and 10 µM in 3T3-L1 adipocytes. Oil Red O staining revealed that treatment with ASC at a concentration of 1 µM significantly reduced the accumulation of lipid in droplets vs. DC cells (Figure 1D,E). Quantitative analysis confirmed that treatment with ASC markedly reduced the intracellular triglyceride (TG) content of the 3T3-L1 adipocytes on day 8 of differentiation. In contrast, treatment with rosiglitazone (Rosi), a negative control involved in promoting adipocyte differentiation, resulted in significantly higher TG content, highlighting its potent adipogenic effect (Figure 1F). Furthermore, the lipid content was significantly lower in cells treated with 10 µM ASC, illustrating the compound’s potent inhibitory effect on lipid accumulation in adipocytes.

### 2.2. ASC Affects the Expression of Key Transcriptional Regulators of Adipogenesis in 3T3-L1 Cells

**Figure 2:**
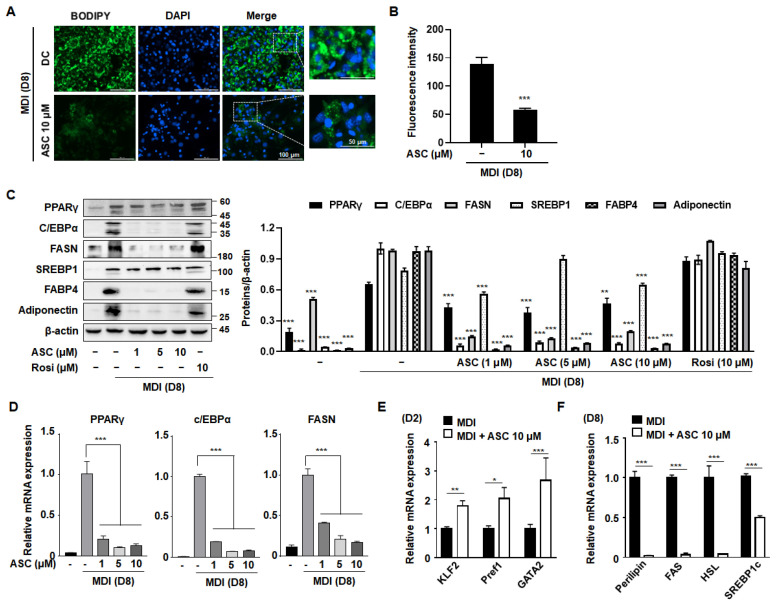
Effect of ASC on lipid accumulation and lipogenic gene expression in 3T3-L1 cells. (**A**) Representative image of BODIPY-stained lipid droplets in DC- or ASC (10 µM)-treated mature 3T3-L1 adipocytes. (**B**) Densitometric analysis of each BODIPY fluorescence ratio was performed using ImageJ (version 2018). (**C**) Two-day post-confluency preadipocytes were incubated with differentiation medium in the presence of ASC (0, 1, 5, and 10 µM). Western blotting of the cells treated with a series of doses of ASC as indicated for PPARγ, C/EBPα, FASN, SREBP1, FABP4, and adiponectin. Protein expression was quantified using ImageJ software and normalized against β-actin as a loading control. (**D**) The expression of PPARγ, C/EBPα, and FASN was evaluated by qRT-PCR with specific primer pairs on D8. (**E**) The expression of KLF2, Pref1, and GATA2 was evaluated by qRT-PCR on D2. (**F**) The expression of Perillipin, FAS, HSL, and SREBP1c was evaluated by qRT-PCR on D8. The relative qRT-PCR values were corrected to expression levels and normalized with respect to the control. Data represent the mean ± SD of three independent experiments. * *p* < 0.05, ** *p* < 0.01, *** *p* < 0.001 compared to the DC.

To evaluate the anti-adipogenic potential of ASC, representative images of BODIPY-stained lipid droplets in mature 3T3-L1 adipocytes treated with ASC at 10 µM were compared with those of DC cells. ASC caused a clear reduction in both the size and number of lipid droplets (Figure 2A,B), confirming that ASC inhibits adipocyte maturation and lipid storage.

To explore the mechanisms underlying this reduction in intracellular triglyceride content during adipocyte differentiation, we measured the protein expression levels of key adipogenic transcription factors. Western blot analysis showed that ASC treatment (at 1, 5, or 10 µM) caused the downregulation of adipogenic proteins, including PPARγ, C/EBPα, FAS, SREBP1, fatty acid binding protein 4 (FABP4), and adiponectin (Figure 2C,D). In addition, the analysis of the mRNA expression of these adipogenic regulators, including PPARγ, C/EBPα, and FAS, during adipocyte differentiation revealed similar significant effects. Although the differentiation medium substantially increased the expression of these adipogenic proteins in 3T3-L1 cells, ASC treatment markedly reduced their mRNA and protein expression, as shown in Figure 2D. This suggests that ASC effectively disrupts adipocyte maturation by reducing the expression of key mediators at the mRNA level.

Next, to determine whether ASC affects adipocyte differentiation at an early stage, we assessed the effects of ASC on the expression of anti-adipogenic genes, such as those encoding Kruppel-like transcription factor 2 (Klf2), preadipocyte factor 1 (Pref1), and GATA-binding protein 2 (Gata2). ASC increased the mRNA expression of these genes on day 2 (D2) of differentiation induction, indicating that ASC has an effect early in adipogenesis (Figure 2E). Moreover, on D8, the expression levels of lipogenic genes were found to be much lower in ASC-treated cells, as shown in Figure 2F. Thus, ASC not only reduces the expression of key adipogenic genes, but also significantly increases the expression of anti-adipogenic genes and markedly reduces the expression of lipogenic genes, thereby reducing lipid accumulation in maturing adipocytes.

### 2.3. ASC Affects the Cell Cycle and Transcriptional Regulation during the Differentiation of 3T3-L1 Cells

**Figure 3:**
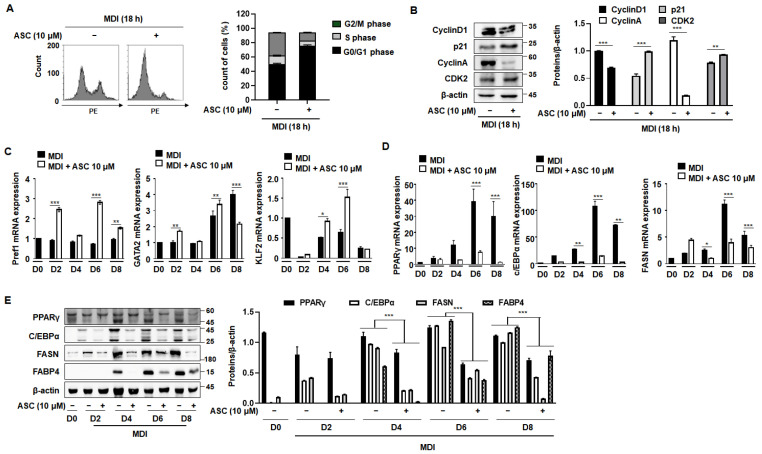
Effect of ASC on MCE and adipogenic differentiation in 3T3-L1 cells. Cells were differentiated according to the standard protocol. (**A**) Flow cytometry analysis of cells treated with MDI and 10 µM ASC for 18 h. (**B**) Cellular extracts were isolated and analyzed for the expression of the proteins indicated by Western blotting. Protein expression was quantified using ImageJ software and normalized against β-actin as a loading control. (**C**) Two-day post-confluency preadipocytes were incubated with differentiation medium in the presence of 10 µM ASC for 48 h. 3T3-L1 cells were subjected to adipocyte differentiation and harvested on day 0, 2, 4, 6, and 8 for qRT-PCR. mRNA levels of anti-adipogenesis-associated genes (Pref1, GATA2, and KLF2). (**D**) Protein levels of pre-adipogenesis-associated genes (PPARγ, c/EBPα, FASN, and FABP4). (**E**) mRNA levels of pre-adipogenesis-associated genes (PPARγ, c/EBPα, and FAS). Data represent the mean ± SD of three independent experiments. * *p* < 0.05, ** *p* < 0.01, *** *p* < 0.001 compared to the DC.

During the initial phase of 3T3-L1 preadipocyte differentiation, cells undergo two sequential mitotic cycles, known as MCE, which are critical for the activation of adipogenic genes [18]. Typically, when growth-arrested post-confluence 3T3-L1 preadipocytes are stimulated with an MDI mixture, they transition from the G0/G1 phase to the S phase within the subsequent 2 days [19]. As shown in Figure 3A, approximately 50% of the cells were in the S and G2/M phases after 18 h of treatment with MDI. However, treatment with ASC prevented this progression to the S and G2/M phases, with the cells predominantly being maintained in the G0/G1 phase. Further investigations were then conducted to better understand how ASC influences cell cycle dynamics during adipocyte differentiation. An analysis of cellular extracts revealed that ASC reduced the expression of the key proteins that regulate cell cycle progression, such as cyclin D1, cyclin A, and cyclin-dependent kinase 2 (CDK2), while significantly preserving the stability of the CDK inhibitor p21 (Figure 3B). These findings were obtained using both FACS analysis and Western blotting. Thus, ASC reduces the expression of S phase proteins and causes cell cycle arrest at the G0/G1 checkpoint.

We next assessed the effects of ASC on the expression of adipogenic and anti-adipogenic genes throughout the differentiation process, from D0 to D8. ASC treatment increased the expression of anti-adipogenic genes, including Pref1, GATA2, and KLF2, and this effect persisted until D6 (Figure 3C). By contrast, key mediators of adipogenesis, such as PPARγ, C/EBPα, and FAS, showed increases in their mRNA level with the progression of differentiation, and ASC treatment significantly reduced their expression (Figure 3D). In addition, the protein expression of these mediators was markedly reduced by ASC treatment, consistent with the mRNA level data (Figure 3E). ASC also reduced the expression of C/EBPδ without affecting C/EBPβ, a key transcription factor in the early stage of adipogenesis (Supplementary Figure S1). These results collectively suggest that ASC not only impedes the MCE that is necessary for the early stage of adipocyte differentiation, but also affects the expression of key transcriptional regulators, thereby inhibiting the differentiation of preadipocytes into mature adipocytes. This dual mechanism of action highlights the potential of ASC as a therapeutic agent that targets adipogenic pathways.

### 2.4. ASC Regulates Β-Catenin Expression and Inhibits Adipogenesis in MDI-Treated 3T3-L1 Cells

**Figure 4:**
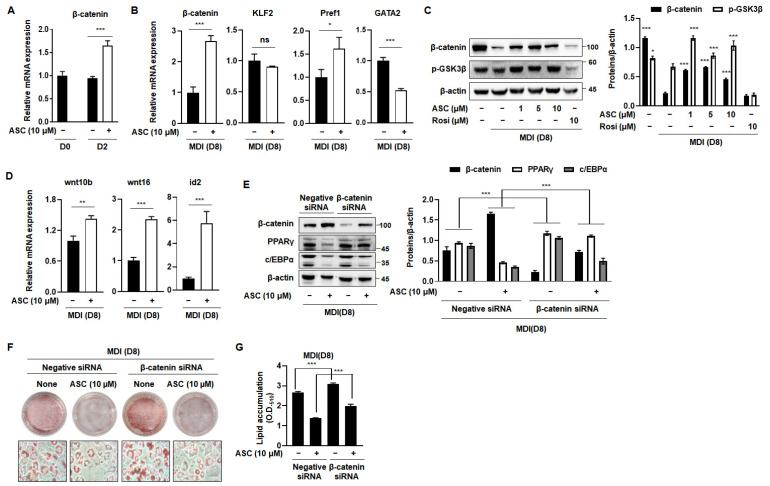
Activation of Wnt/β-catenin signaling by ASC blocked adipogenesis. (**A**) mRNA expression levels of β-catenin in 3T3-L1 adipocytes treated with ASC (10 µM) at days 2 and 8. (**B**) The expression of β-catenin, KLF2, Pref1, and GATA2 was evaluated by qRT-PCR with specific primer pairs on D8. (**C**) Representative Western blot images and quantification of β-catenin and p-GSK3β protein levels in fully matured 3T3-L1 adipocytes. Protein expression was quantified using ImageJ software and normalized against β-actin as a loading control. (**D**) The expression of Wnt10b, Wnt16, and id2 was evaluated by qRT-PCR with specific primer pairs on D8. (**E**) 3T3-L1 cells were transfected with control siRNA or β-catenin siRNA, and then treated with ASC (10 µM) and harvested on day 8. Cells were analyzed by Western blotting for the β-catenin, PPARγ, and c/EBPα. β-actin was used as a loading control. (**F**) Representative images of 3T3-L1 cells were transfected with control siRNA or β-catenin siRNA, and then treated with ASC (10 µM) and stained with Oil Red O on day 8. (**G**) Quantification of intracellular lipid content. Data represent the mean ± SD of three independent experiments. ns: not significant, * *p* < 0.05, ** *p* < 0.01, *** *p* < 0.001 compared to the DC.

The Wnt/β-catenin signaling pathway is a pivotal negative regulator of the transcription factors that are essential for adipocyte differentiation [8]. The activation of Wnt signaling reduces the mRNA and protein expression of C/EBPα and PPARγ, which are crucial for terminal differentiation [20]. Therefore, we investigated the effect of ASC on the expression of β-catenin, a key component of this pathway, which directly regulates adipogenesis. ASC at a concentration of 10 µM significantly increased the mRNA expression of β-catenin in MDI-treated 3T3-L1 cells on D2 (Figure 4A). In addition, we evaluated the effects of ASC on the mRNA expression of anti-adipogenic genes, including β-catenin, Klf2, Pref1, and Gata2, on D8. ASC significantly increased the mRNA expression of β-catenin, but not that of the other genes, on the D8 (Figure 4B). To further explore the impact of ASC on adipogenesis, we analyzed the protein levels of β-catenin and p-Glycogen synthase kinase 3β (GSK3β) in 3T3-L1 cells treated with 1, 5, and 10 µM ASC. The treatment of MDI markedly reduced the protein expression of β-catenin and p-Glycogen synthase kinase 3β (GSK3β) compared to preadipocytes. However, ASC treatment significantly elevated the expression of these proteins (Figure 4C). In addition, ASC increased the mRNA levels of the Wnt signaling pathway including wnt10b, wnt16, and id2 (Figure 4D). These results suggest that the anti-adipogenesis effects of ASC related to the increasing of Wnt/β-catenin signaling pathway on the terminal differentiation.

To confirm that ASC inhibits adipogenesis through the Wnt signaling pathway, we measured the expression of adipogenesis-related genes in 3T3-L1 cells transfected with β-catenin siRNA. Transfection with β-catenin siRNA reduced the protein expression of β-catenin and attenuated the ASC-induced increase in β-catenin expression. This reduction was also accompanied by an increase in the protein expression of PPARγ and C/EBPα (Figure 4E). As expected, β-catenin siRNA treatment increased lipid droplet formation vs. differentiation control cells. It also inhibited the reduction in lipid droplet size caused by ASC (Figure 4F,G). Overall, these results suggest that ASC inhibits adipogenesis in 3T3-L1 adipocytes by activating the Wnt/β-catenin signaling pathway.

### 2.5. ASC Ameliorates HFD-Induced Obesity

**Figure 5:**
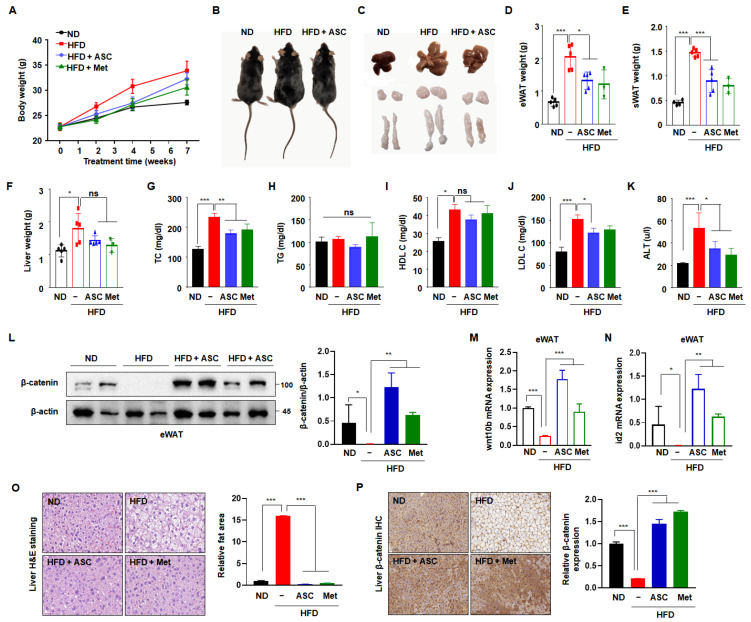
Effect of ASC on HFD-induced obesity. (**A**) Weekly change in total body weight and (**B**) representative appearance of mice fed with normal diet (ND, *n* = 5), high-fat diet (HFD, *n* = 5), ASC (5 mg/kg, *n* = 5), and metformin (Met, 200 mg/kg, *n* = 3). (**C**) Representative appearance of liver, inguinal subcutaneous (iWAT), and epididymal fat tissue (eWAT). (**D**) Changes in liver and (**E**) eWAT and (**F**) iWAT weight. (**G**–**K**) Plasma level of TC, TG, HDL cholesterol (HDL-C), LDL cholesterol (LDL-C), and alanine aminotransferase (ALT). (**L**) Representative Western blot images and quantification of β-catenin protein levels in eWAT are shown. Protein expression was quantified using ImageJ software and normalized against β-actin as a loading control. (**M**,**N**) The expression of Wnt10b and id2 was evaluated in eWAT by qRT-PCR using specific primer pairs. (**O**) H&E-stained images of liver tissue. (**P**) β-catenin expression of the liver tissues by immunohistochemistry, 20×. Data represent the mean ± SD of three independent experiments. ns: not significant, * *p* < 0.05, ** *p* < 0.01, *** *p* < 0.001 compared to the HFD.

To validate the results of the in vitro experiment, C57BL/6J mice were fed an HFD for 10 weeks to establish diet-induced obesity and permit the investigation of the effect of ASC to prevent obesity. After the mice had acclimated to the HFD for 1 week, ASC (5 mg/kg) was administered intraperitoneally. Metformin, a conventional anti-diabetic agent, was used as a positive control. The mice were then sacrificed after 11 weeks. The body mass of the HFD group was higher than that of the ND group. However, both ASC and metformin tended to reduce the body mass of the HFD-fed mice (Figure 5A). As shown in Figure 5B,C, the masses of the body, liver, subcutaneous fat depot, and epididymal fat depot were significantly higher in the HFD group than in the ND group, but these differences were attenuated by ASC treatment. A more significant reduction in fat content occurred in the epididymal and subcutaneous fat depots, and ASC had the larger effect, except in the liver (Figure 5D–F). Furthermore, ASC significantly reduced the serum total cholesterol and LDL-cholesterol concentrations the HFD-fed mice, but did not affect the TG concentration (Figure 5H–K). The serum activity of the liver-specific enzyme ALT was >230% higher in the HFD group than in the ND group, with levels reaching approximately 53 U/L. However, the serum ALT activities of mice treated with ASC were significantly lower (37 U/L), suggesting that ASC mitigates the hepatotoxic effect of HFD in mice (Figure 5G).

To determine whether ASC inhibits adipogenesis through increased β-catenin activation in vivo, β-catenin protein expression was measured in eWAT. As expected, the β-catenin expression was significantly lower in the HFD group compared to the ND group. ASC treatment notably increased β-catenin expression (Figure 5L). In addition, ASC increased the mRNA expressions of wnt10b and id2 in eWAT, which were reduced by HFD (Figure 5M,N). These results suggest that the anti-obesity effects of ASC relate to the upregulation of the Wnt/β-catenin signaling pathway. To investigate whether the higher liver mass of the HFD mice was the result of the hypertrophy or hyperplasia of adipocytes, liver tissue sections were stained with hematoxylin and eosin. The sizes of adipocytes within the liver significantly differed between the ND and HFD groups, and the adipocytes in the ASC and metformin groups showed smaller adipocytes compared to the HFD group (Figure 5L). We hypothesized that the expression of β-catenin would be higher in the ASC group, and indeed, the HFD group showed no β-catenin-associated immunostaining in the liver tissue, whereas abundant immunostaining was present in the ASC and metformin groups (Figure 5M). Overall, these results suggest that ASC mitigates HFD-induced obesity by reducing body and fat mass, reducing serum cholesterol concentrations, and also perhaps by modulating β-catenin expression in the liver.

## 3. Discussion

Various natural products have been investigated for their potential to inhibit adipogenic differentiation and reduce fat accumulation, offering a safer alternative to conventional anti-obesity drugs, which have significant side effects [21,22,23]. Previous studies have shown that plant extracts and bioactive compounds can reduce the expression of key adipogenic transcription factors, such as PPARγ and C/EBPα, and also inhibit signaling pathways, such as the PI3K/AKT pathway, thereby having anti-adipogenic effects [22]. Compounds such as rebaudioside A, sativoside, and theasaponin E1 have been shown to have anti-adipogenic and anti-lipogenic effects, thus demonstrating their potential as anti-obesity treatments [24]. These findings indicate the importance of understanding the mechanisms of adipogenesis and interrogating natural products for their ability to safely ameliorate or prevent obesity. Therefore, in the present study, we explore the potential of ASC, a natural product, as an anti-obesity agent. We evaluated the anti-obesity effects of ASC in both 3T3-L1 adipocytes, a mouse preadipocyte cell line, and in C57BL/6J mice with HFD-induced obesity.

In a previous study, we demonstrated that the ASC derivative, MAC, inhibited the differentiation of 3T3-L1 preadipocytes [25]. However, the current study shows contrasting results, with ASC exhibiting more potent inhibitory effects on adipogenesis compared to MAC. This discrepancy can be attributed to differences in the timing and frequency of compound administration. In the previous work, MAC was administered repeatedly, and the differentiation medium was replaced each time (days 0, 2, and 4). This repeated dosing may have contributed to the inhibitory effects observed with MAC. In the present study, both ASC and MAC were applied only once, at the initiation of differentiation with MDI medium, without subsequent treatments. Under these conditions, MAC failed to inhibit lipid accumulation (Figure 1B,C), while ASC demonstrated significant inhibition with a single treatment. This suggests that ASC’s inhibitory effects on adipogenesis are more robust and do not require repeated dosing, unlike MAC. These findings highlight the distinct bioactivity of ASC and underscore its potential as a stronger candidate for further anti-obesity investigations.

Adipogenesis plays a crucial role in determining the number of adipocytes and influencing lipid storage capacity, rendering it a key target in anti-obesity strategies [22,26,27]. We have shown that ASC significantly reduces the expression of key adipogenic mediators, such as PPARγ, C/EBPα, and FAS, which are essential for the terminal differentiation of adipocytes and lipid accumulation within these cells, over an 8-day differentiation period. In addition, ASC increased the expression of anti-adipogenic genes, such as Klf2, Pref1, and Gata2, during the early stages of adipocyte differentiation. Klf2 is known to be highly expressed in 3T3-L1 preadipocytes, with its expression decreasing rapidly as differentiation progresses [28]. In this study, the upregulation of Klf2 observed in Figure 2E during the early stages of differentiation (Day 2) is consistent with ASC’s role in inhibiting adipogenesis, as Klf2 is maintained at higher levels to suppress differentiation. In contrast, the absence of Klf2 upregulation in Figure 4B at the later stage (Day 8) reflects the natural downregulation of Klf2 as preadipocytes transition into mature adipocytes. This temporal shift in Klf2 expression aligns with its established role in inhibiting adipogenesis during the early stages but diminishing as differentiation progresses [28]. Numerous phytochemicals have been reported to inhibit adipogenesis by targeting key steps, such as proliferation, cell cycle regulation, and the transcription factors involved in early adipocyte differentiation [27,28,29]. The present results suggest that the inhibitory effect of ASC on adipogenesis is associated with the upregulation of anti-adipogenic genes during the early stages of differentiation.

In the initial stage of adipocyte differentiation, the regulation of MCE is crucial. When growth-arrested post-confluent 3T3-L1 preadipocytes are stimulated to differentiate by MDI, they undergo two sequential mitotic cycles within the subsequent 2 days [30]. MCE is essential for the transition of preadipocytes to mature adipocytes, because this involves the critical phase of cell cycle re-entry and proliferation [11]. By maintaining preadipocytes in the G0/G1 phase and preventing their progression into the S phase, ASC disrupted the early critical phase of adipocyte maturation. This disruption was accompanied by a decrease in the expression of cyclins and CDKs, which are essential for cell cycle progression, but a stabilization of the inhibitor p21. These findings indicate that a key mechanism by which ASC inhibits adipocyte differentiation is the suppression of MCE.

The Wnt/β-catenin pathway is well established as a key regulator in cell fate determination, particularly in mesenchymal stem cells (MSCs) where it inhibits adipogenesis while promoting osteogenesis [31]. Active Wnt signaling has been shown to maintain MSCs in an undifferentiated state by suppressing key adipogenic transcription factors such as PPARγ and C/EBPα, which are essential for terminal differentiation into mature adipocytes [32,33]. Our findings are consistent with previous studies that demonstrated the inhibitory role of the Wnt/β-catenin pathway during the early stages of adipogenesis. Specifically, ASC treatment significantly reduced intracellular lipid accumulation and downregulated the expression of adipogenic markers, including PPARγ, C/EBPα, and FAS. This is consistent with the inhibition of Wnt signaling in adipogenesis, where reduced Wnt activity allows preadipocytes to proceed with terminal differentiation. However, in the presence of ASC, the Wnt/β-catenin pathway appears to remain active, thereby preventing the initiation of adipogenesis. The results of β-catenin knockdown further confirmed the interaction between ASC and the Wnt/β-catenin pathway, and are therefore consistent with ASC obtaining its anti-obesity effects through an upregulation of β-catenin. Our findings suggest that while β-catenin plays a significant role in the effects of ASC, the inhibition of MCE provides an additional, crucial pathway through which ASC reduces lipid accumulation. This comprehensive approach not only reinforces the complexity of the mechanisms underlying ASC’s anti-adipogenic effects, but also underscores the potential of targeting multiple facets of adipocyte differentiation in obesity management.

In addition, the animal experiment using a mouse model of HFD-induced obesity demonstrated that ASC reduces the body masses of mice and the sizes of the subcutaneous and epididymal fat depots. Although a significant reduction in liver mass was not identified, ASC reduced the serum ALT activities and hepatic steatosis of the mice. Interestingly, ASC induced an increase in hepatic β-catenin expression in the mice, which suggests that it may modulate the Wnt/β-catenin pathway, not only in adipose tissue, but also in the liver. These findings highlight the potential value of ASC to treat metabolic disorders associated with HFD-induced obesity.

In conclusion, this was the first study to explore the anti-obesity effects of ASC. We have demonstrated that ASC has anti-obesity effects by inhibiting MCE and increasing β-catenin expression, thereby influencing the early stage of adipocyte differentiation. Through both in vitro and in vivo experiments, we have shown that ASC has considerable potential as an anti-obesity drug. These findings should encourage further exploration into the mechanisms of the effects of ASC and its potential applications in obesity management.

## 4. Materials and Methods

### 4.1. Cells and Materials

3T3-L1 preadipocyte cells were obtained from the American Type Culture Collection (Manassas, VA, USA). The cells were incubated in DMEM (HyClone; GE Healthcare Life Sciences, Logan, UT, USA) supplemented with 10% bovine serum (BS, Gibco BRL, Grand Island, NY, USA), penicillin–streptomycin, and 1% antibiotic mixture in an atmosphere of 5% CO_2_ at 37 °C. The medium was subsequently changed every 2 days with DMEM containing 10% BS. Adipocyte differentiation was initiated by replacing the medium with DMEM supplemented with 10% fetal bovine serum (FBS, Gibco BRL), 0.1 mM isobutylmethylxanthine (IBMX, Sigma, St. Louis, MO, USA), 1 mM dexamethasone (Sigma), and 5 µg/mL insulin (Sigma, human, Roche) on day 0, two days after the cells had reached confluence. ASC and associated compounds were added only once at the time of MDI treatment (day 0–2), and the medium was replaced with DMEM containing 10% FBS and 5 µg/mL insulin every 2 days without further addition of the compounds (day 3–4). Then, the medium was changed to only DMEM containing 10% FBS for the last 4 days (day 5–8). This single treatment protocol was used to evaluate the inhibitory effects of ASC on adipocyte differentiation and lipid accumulation. Morphological changes began on day 4, progressing into mature adipocytes, and the cells were incubated for a total of 8 days. ASC (≥98% HPLC) was obtained from Chugai Pharmaceutical (Tokyo, Japan). A stock solution of ASC (10 mM) was prepared in DMSO, stored at −20 °C, and diluted in medium prior to use. Densitometric analysis was performed on scanned Western blot and IHC images using ImageJ software (version 2018; National Institutes of Health, Bethesda, MD, USA).

### 4.2. Cell Viability Assay

Preadipocytes were seeded in 96-well plates at a density of 2 × 10^4^ cells/well in 100 µL of complete medium and incubated at 37 °C in an atmosphere of 5% CO_2_ for 24 h. They were then exposed to various concentrations of ASC for 48 h. At the end of this treatment period, 10 µL of MTT (3-(4,5-Dimethylthiazol-2-yl)-2,5-Diphenyltetrazolium Bromide, Invitrogen) was added to each well. The plates were further maintained at 37 °C for 4 h. Subsequently, the formed formazan was dissolved with DMSO. The results were measured using a microplate reader (VersaMax microplate reader, Molecular Devices, Sunnyvale, CA, USA) at an absorbance of 540 nm.

### 4.3. Oil Red O Staining Analysis of Adipocytes

Differentiated adipocytes were fixed in 10% formalin for 30 min. After discarding the formalin, the cells were covered with 60% isopropanol and incubated at room temperature for 5 min. A solution of Oil Red O (Sigma, USA) was prepared in a 3:2 ratio with distilled water (DW). This staining solution was applied to the cells for 30 min. After the staining period, the cells were washed several times with DW until the wash water was clear. Lipid droplets were visualized using inverted microscopes (ECLIPSE TS100). To quantify lipid accumulation, the lipids were dissolved in 100% isopropanol, and the absorbance was measured at 480 nm using a microplate reader.

### 4.4. Triglyceride Assay

Triglycerides were extracted from differentiated adipocytes using the PicoSens Triglyceride Quantification Kit (Colorimetric/Fluorometric, BIOMAX, Seoul, Republic of Korea) according to the manufacturer’s instructions. A standard curve was prepared using a 1 mM triglyceride standard, with concentrations ranging from 0 to 8 nmol/well. The glycerol background control was subtracted from results to correct for any baseline interference. Triglyceride levels in the adipocytes were quantified by measuring the absorbance at 570 nm using a microplate reader.

### 4.5. BODIPY Staining Assay

Preadipocytes were seeded in 8-well slides at a density of 5 × 10^4^ cells/well and cultured in a humidified 5% CO_2_ environment at 37 °C. After 8 days of adipogenic culture, the adipocytes were washed with PBS and then stained with 4,4-Difluoro-1,3,5,7,8-Pentamethyl-4-Bora-3a,4a-Diaza-s-Indacene (BODIPY 493/503, Carlsbad, CA, Invitrogen) at 37 °C for 15 min. Following this, the cells were washed twice with PBS and fixed with 4% paraformaldehyde (PFA) (Biosesang, Seongnam, Republic of Korea) for 30 min at room temperature. The cells were then stained with 4′,6-diamidino-2-phenylindole dihydrochloride (DAPI, Sigma, USA). After staining, the cells were washed three times with 0.05% Tween 20 (Sigma, USA) and mounted with Fluorescence Mounting Medium (Dako, Glostrup, Denmark) at room temperature, with the mounting process lasting between 1 h and 1 day. Fluorescent staining was visualized using a BioTek Cytation 1 imaging reader (Agilent, Santa Clara, CA, USA) at a 20× magnification. Densitometric analysis of the captured images was performed using ImageJ software (version 2018; National Institutes of Health, Bethesda, MD, USA).

### 4.6. Real-Time Polymerase Chain Reaction (RT-PCR) Assay

Total RNA was extracted using TRIzol (Bio Science Korea, Daegu, Republic of Korea) following the manufacturer’s protocol. For reverse transcription, cDNA was synthesized from the final 3 µg/µL RNA using Prime Script RT Master Mix (Takara, Japan) according to the manufacturer’s instructions. The cDNA was amplified by real-time PCR using the primers in Table 1. mRNA levels were normalized against β-actin, and the relative levels were measured using the ΔCt method.

### 4.7. Immunoblotting Assay

Preadipocytes were seeded in 6-well plates and 60 mm dishes at a density of 5 × 10^5^ cells/well, and cultured in a humidified environment of 5% at 37 °C. Cells were lysed in RIPA lysis buffer at 4 °C and then centrifuged at 13,000 rpm for 10 min. Then, 20 µg of protein was loaded using acrylamide gradient gel (SDS-PAGE) and transferred to the Immobilon-P membranes (PVDF) (Millipore, Cork, Ireland) and Nitrocellulose blotting membrane (NC). The membrane was blocked with 5% skim milk for 30 min and then incubated with primary antibody overnight at 4 °C. PPARγ (sc-7273), SREBP1 (sc-365513), Cyclin E (sc-198), CDK2 (sc-163), p21 (sc-397), Cyclin A (sc-596), β-catenin (sc-7963), and β-actin (sc-47778) antibodies were purchased from Santa Cruz Biotechnology, Inc. (Dallas, TX, USA). C/EBPα (#8178) and FABP4 (#2120) antibodies were purchased from Cell Signaling Technology, Inc. (Danvers, MA, USA). FAS (A0461), Adiponectin (A21135), Cyclin D1 (A19038), p27 (A0290), HMGB2 (A2973), SPRY1 (A10278), C/EBPβ (A0711), and C/EBPδ (A15261) antibodies were purchased from ABclonal Science, Inc. (Woburn, MA, USA). The membranes were washed three times each for 10 min with TBS-T, and then incubated for 2 h at room temperature with goat anti-mouse IgG (HRP) (#31430) and goat anti-rabbit IgG (HRP) (#65-6120) secondary antibodies diluted to 1:2000 in 2% skim milk. Chemiluminescence (GE Healthcare, Chicago, IL, USA) was used to confirm protein expression, which was detected using the Davinch-Chemi Imaging System (Davinch-K, Seoul, Republic of Korea).

### 4.8. Cell Cycle Analysis

3T3-L1 cells were seeded in 60 mm dishes at a density of 5 × 10^5^ cells/well and cultured in a humidified environment with 5% CO_2_ at 37 °C. Adipocytes at 18 h of differentiation were harvested and centrifuged at 3000 rpm for 3 min. The supernatant was discarded, and the cells were fixed by 80% EtOH. The adipocytes were fixed for 1 h at 4 °C. Subsequently, the fixed cells were stained with propidium iodide (PI) solution (10% NP-40, 10 mg/mL RNase, 10 mg/mL PI stock solution, 0.01 M PBS) for 30 min at 4 °C by pipetting. The adipocyte cell cycle at 18 h was analyzed using flow cytometry (Beckman Coulter, Miami, FL, USA).

### 4.9. siRNA Transfection

The preadipocyte cells were transfected with a final concentration of 50 nmol/L of negative control siRNA (5′-GUUCAGCGUGUCCGGCGAGTT-3′) or β-catenin-specific siRNA duplex (5′-GAAACGGCTTTCAGTTGAG-3′) (Bioneer, Daejeon, Republic of Korea) with Lipofectamine™ RNAiMAX Transfection Reagent (Invitrogen, Waltham, MA, USA) according to the manufacturer’s instructions. After 5–6 h, the medium was replaced with 0.5% bovine serum medium without antibiotics and the cells were maintained for 36–48 h. Finally, the medium was changed to antibiotic-free 10% bovine serum medium, and the cells were maintained.

### 4.10. Animals

C57BL/6J mice (male, 5 weeks old) were purchased from Lab Animal (Seoul, Republic of Korea) and maintained under pathogen-free conditions. After one week of acclimatization, the mice were divided into four experimental groups (*n* = 3–5 per group): a normal diet (ND, 10% kcal from fat, D12450B, Research Diets, Inc., New Brunswick, NJ, USA) group, a high-fat diet (HFD, 60% kcal from fat, D12451, Research Diets, Inc.) group, and two HFD groups that received intraperitoneal (IP) injection of either ASC dissolved in 0.2% Tween 20 or metformin dissolved in PBS. The mice were fed either a normal diet (ND) or a high-fat diet (HFD) for 4 weeks. Starting from the 4th week, the mice received diet and drug injections concurrently until the 11th week, at which point they were sacrificed. Total cholesterol (TC), high-density lipoprotein cholesterol (HDL-C), low-density lipoprotein cholesterol (LDL-C), and alanine aminotransferase (ALT) assays were conducted by Preclina Inc. (Daegu, Republic of Korea) at Kyungpook National University. The animal care and all experimental procedures were approved by and conducted in accordance with the guidelines of the Institutional Animal Care and Use Committee of the Catholic University of Daegu.

### 4.11. Immunohistochemistry (IHC)

Paraffin-embedded epididymal white adipose tissue (eWAT) and subcutaneous white adipose tissue (sWAT) were mounted on microscope slides. The tissue sections were maintained with β-catenin antibodies at 4 °C overnight. Secondary antibodies were incubated at 37 °C for 30 min. 3,3′-diaminobenzidine tetrahydrochloride was used as the coloring reagent, and hematoxylin was used as the counterstain. Tumor sections were observed using a Pannoramic MIDI slide scanner (3DHISTECH, Budapest, Hungary).

### 4.12. Statistical Analysis

All in vitro tests were performed in triplicate for each independent experiment. The values of *** *p* < 0.001, ** *p* < 0.01, and * *p* < 0.05 indicate statistical significance between the experimental and control values. Errors between the experimental and control values were calculated using a one-way ANOVA in Prism.

## Figures and Tables

**Table 1:** Primers used for RT-PCR analysis in the present study.

Genes	Sense Primer (5′–3′)	Antisense Primer (5′–3′)
PPAR-γ	ATCAAAGTAGAACCTGCATC	ACCCTTGCATCCTTCACAAG
C/EBP-α	GAACAGCAACGAGTACCGGGT	CCCATGGCCTTGACCAAGGAG
pref-1	TGGCTGTGTCAATGGAGTCTGC	CCACGCAAGTTCCATTGTTGGC
GATA2	CTTCAACCATCTCGACTCGCAG	GCAACAAGTGTGGTCGGCACAT
β-catenin	GTTCGCCTTCATTATGGACTGCC	ATAGCACCCTGTTCCCGCAAAG
Wnt10b	ACCACGACATGGACTTCGGAGA	CCGCTTCAGGTTTTCCGTTACC
Wnt16	CCCTCTTTGGCTATGAGCTGAG	GGTGGTTTCACAGGAACATTCGG
Tcf7l2	CGCTGACAGTCAACGCATCTATG	GGAGGATTCCTGCTTGACTGTC
Axin2	ATGGAGTCCCTCCTTACCGCAT	GTTCCACAGGCGTCATCTCCTT
Nkd1	GCCTGAGAAGATTGACAGCCTAG	CTCCACAGAGACATCACACTGC
β-actin	AGGCCCAGAGCAAGAGAGGTA	CCATGTCGTCCCAGTTGGTAA

## Data Availability

Data is contained within the article or Supplementary Material.

## Supplementary Materials

The following supporting information can be downloaded at: https://www.mdpi.com/article/10.3390/ijms251810226/s1.
