# Supplementary figures and images for: Ascochlorin Attenuates the Early Stage of Adipogenesis via the Wnt/β-Catenin Pathway and Inhibits High-Fat-Diet-Induced Obesity in Mice
Source: Int J Mol Sci. 2024 Sep 23;25(18):10226. doi: 10.3390/ijms251810226 (PMC11432539; doi:10.3390/ijms251810226)

Supplementary data. Figure 1.

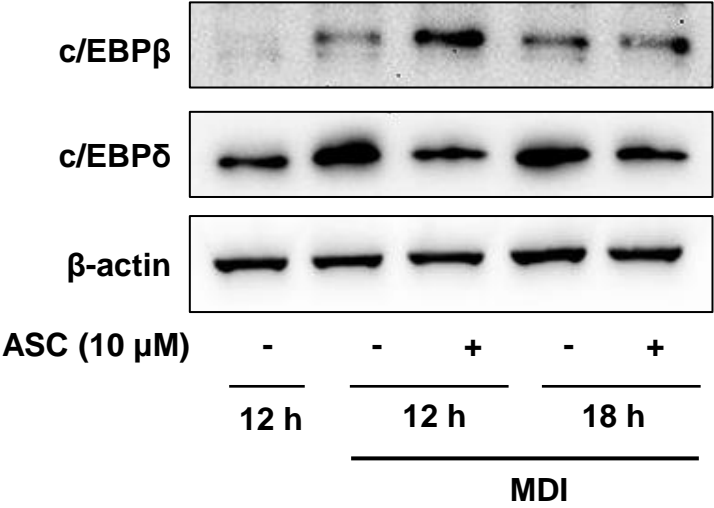

## Supplementary data. Raw Data

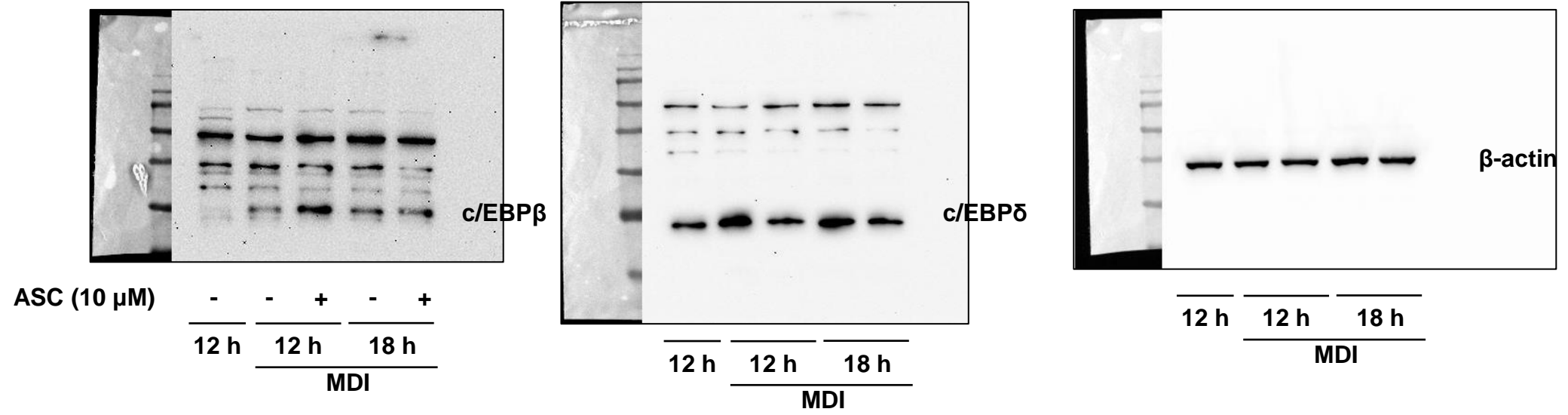

Supplement: Supplementary file 1 [file ijms-25-10226-s001.zip › ijms-3200623-supplementary.pdf]
